# Lupeol ameliorates LPS/D-GalN induced acute hepatic damage by suppressing inflammation and oxidative stress through TGFβ1-Nrf2 signal pathway

**DOI:** 10.18632/aging.202409

**Published:** 2021-03-11

**Authors:** Sha Huang, Chan Mo, Ting Zeng, Yuqi Lai, Chuying Zhou, Shunwen Xie, Limei Chen, Yuhua Wang, Yuyao Chen, Shaohui Huang, Lei Gao, Zhiping Lv

**Affiliations:** 1 School of Traditional Chinese Medicine, Southern Medical University, Guangzhou 510515, Guangdong, China; 2 The Key Laboratory of Molecular Biology, State Administration of Traditional Chinese Medicine, School of Traditional Chinese Medicine, Southern Medical University, Guangzhou 510515, Guangdong, China; 3 Guangdong Provincial Key Laboratory of Shock and Microcirculation, Southern Medical University, Guangzhou 510515, Guangdong, China; 4 Zhujiang Hospital, Southern Medical University, Guangzhou 510280, Guangdong, China

**Keywords:** lupeol, TGFβ1, Nrf2, acute liver injury, inflammation

## Abstract

Acute hepatic damage is a severe condition characterized by inflammation and oxidative stress, which is a serious threat to people's life and health. But there are few effective treatments for acute liver injury. Therefore, safe and effective therapeutic approaches for preventing acute liver damage are urgently needed. Lupeol is a natural compound, which has significant antioxidant and anti-inflammatory properties in liver disease. However, the protective mechanism of lupeol against acute liver injury remains unclear. Here, zebrafish and mutant mice were utilized to investigate the protective effects of lupeol against lipopolysaccharide (LPS)/ D-galactosamine(D-GalN) -induced liver injury and the underlying mechanisms. We found that pretreatment with lupeol attenuated the LPS/D-GalN-induced liver injury by decreasing the infiltration of inflammatory cells and reducing pro-inflammatory cytokines. We also demonstrated that lupeol could protect injured liver from oxidative stress by downregulating the expression of TGFβ1 and upregulating Nrf2. Notably, our experimental results provided the support that lupeol effectively protected against LPS/D-GalN-induced acute liver injury via suppression of inflammation response and oxidative stress, which were largely dependent on the upregulation of the Nrf2 pathway via downregulating TGFβ1.

## INTRODUCTION

Acute liver injury (ALI) is a life-threatening disease, if not treated in time, it will eventually lead to acute liver failure when the extent of hepatocyte death exceeds the liver's regenerative capacity, and its pathogenesis involves direct damage and immune-mediated injury [1]. Oxidative stress plays a key role in hepatocyte injury because the liver is a major organ invaded by reactive oxygen species and reactive nitrogen species (RNS) [2–4]. Additionally, acute hepatic damage is closely related to inflammation, as inflammatory immune responses characterized by the expression of proinflammatory mediators such as TNF-α and extensive immune cell infiltration in the liver, eventually result in hepatic apoptosis [5]. Although the pathogenic mechanism and factors associated with ALI have been widely reported, the details of liver injury and the drugs effective for treating liver injury remain poorly understood.

Oxidative stress refers to the imbalance between ROS / RNS production from aerobic metabolism and the elimination of antioxidant defense [6]. The antioxidant defense system consists of glutathione (GSH) and its synthesis, phase II detoxification enzyme and active oxygen deactivation enzyme, which play a key role in protecting cells from oxidative damage [7]. Nrf2, the transcription factor nuclear factor-erythroid 2 related factor 2, plays a protective role in GSH synthesis, antioxidant stress system, conjugation, transport and excretion of the metabolites and serves as a pleiotropic target resistant to hepatic damage [8]. It has been reported that Nrf2 induced glutamic acid cysteine ligase gene expression contributes to GSH synthesis and meliorates NAPQI induced hepatotoxicity [9]. Furthermore, activation of Nrf2 decreases acetaminophen (AA) - sulfate formation and enhances elimination of AA- glucuronide due to increased expression of Mrp3 in Keap1-kd mice [10]. Another study finds that transforming growth factor β1 (TGFβ1) induces HO-1 protein expression and enhanced nuclear accumulation of Nrf2 in Human aortic smooth muscle cells (HAoSMC), which also demonstrates that Nrf2-ARE pathway represents a novel target for TGF-β1 in human vascular smooth muscle cells (SMC) [11]. However, the relationship between Nrf2 and TGFβ1 in the regulation of liver diseases is rarely reported.

TGFβ1, a member of the TGFβ family of growth and differentiation factors, controls cell differentiation and proliferation and plays key roles in skeletal diseases, fibrosis, and cancer [12]. TGFβ1 transduces its signal by directly binding TGFβ receptor 2 (TGFβr2) to form a constitutively active kinase and then recruiting TGFβ receptor 1 (TGFβr1) into a heterotetramer receptor complex, ultimately resulting in the phosphorylation of SMAD2 and SMAD3 [13]. Previous studies have shown that TGFβ1 increased in the liver and serum of mice or rats during liver failure [14, 15]. TGFβ1 was markedly elevated in both the liver tissue and the plasma in patients with acute liver failure [16, 17], indicating that TGFβ1 may play a vital role in the regulation of ALI. Upon this, candidate compounds with multi-effects on anti-inflammation, anti-oxidative stress and TGFβr1 and Nrf2 signaling pathway regulation seem to be a more suitable treatment strategy for ALI.

Lupeol, as a natural triterpenoid, is widely found in fruits such as strawberry, mango, grape and olive and vegetables such as white cabbage and green pepper [18]. As a pentacyclic triterpene, lupeol has been proven to have antioxidative, anti-inflammatory, and skin healing-promoting functions and to have inhibitory effects on breast cancer, prostate cancer and mouse melanoma [19, 20]. Besides, lupeol possesses many potential liver-protective effects. A previous study showed that lupeol has a protective effect on aflatoxin B1-induced peroxidative hepatic damage in rats and is as effective as silymarin [21]. Furthermore, other studies have found that lupeol is effective in combating oxidative stress-induced liver injury [22–24]. Thus, in view of the anti-oxidative and anti-inflammatory effects of lupeol, it may be an effective therapy for acute hepatic damage.

Therefore, we investigated the effects of lupeol on ALI in mice and zebrafish, as well as the related mechanisms. We found that lupeol relieved LPS/D-GalN-induced ALI by inhibiting hepatocyte inflammation, hepatic apoptosis and oxidative stress in the liver. Moreover, lupeol cured ALI by decreasing the expression of TGFβr1 and increasing the expression of Nrf2.

## RESULTS

### Lupeol alleviates LPS-induced liver injury in zebrafish

**Figure 1:**
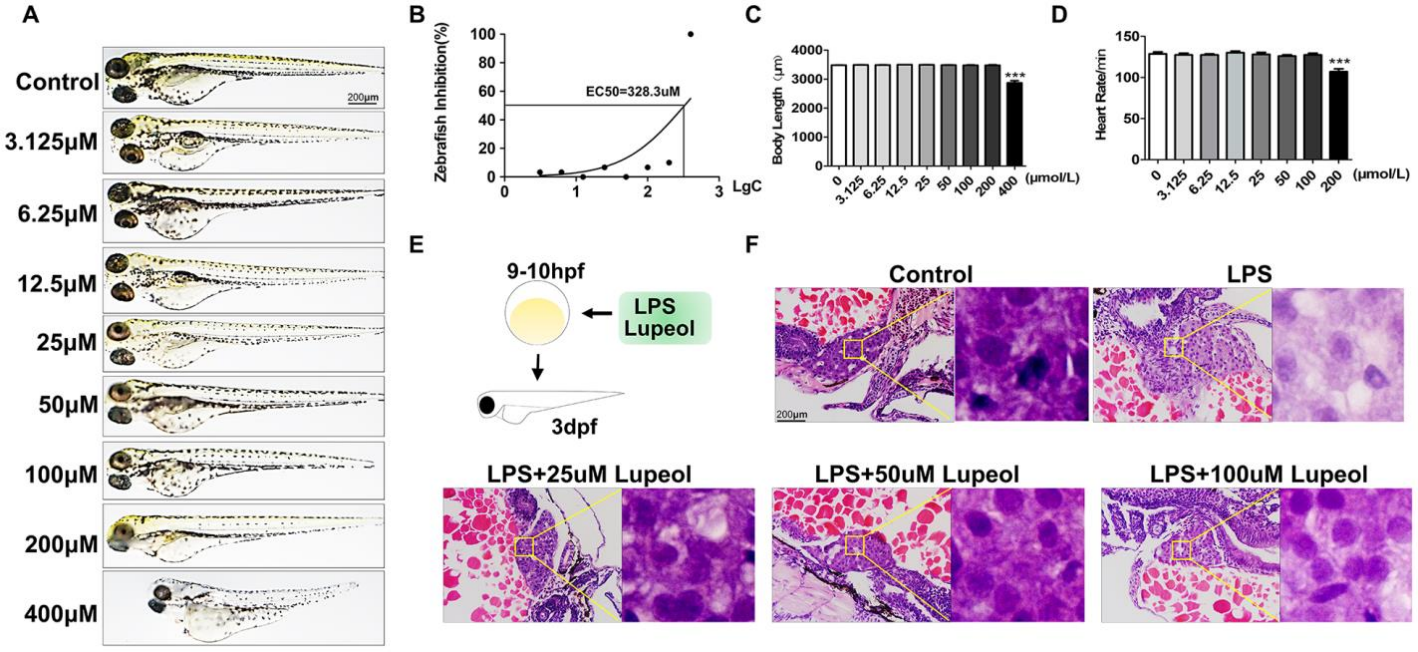
**Lupeol attenuates liver injury induced by LPS in zebrafish.** (**A**–**D**) Zebrafish larvae were treated with different concentrations of lupeol (0, 3.125, 6.25,12.5,25,50,100,200, and 400μmol) to observe changes in zebrafish morphology, survival rate, body length and heart rate. N=20, data are expressed as the mean ± SEM, *P<0.05, **P<0.01, ***P<0.001, control group vs administration group. (**E**) Schematic diagram of treatment on zebrafish. (**F**) H&E staining of the liver in zebrafish larva. Magnification, 400×, bar=200μm.

First, we used the survival rate, body length, heart rate and morphological changes of zebrafish embryos to investigate the toxicology of lupeol. Zebrafish embryos (3 dpf) were treated with lupeol for 72 h. At 400 μM lupeol, the zebrafish larvae had abdominal swelling and a shorter body length (Figure 1A and Supplementary Figure 1A), indicating that 400 μM lupeol did have toxic effects on the developmental stages of zebrafish. Notably, 400 μM lupeol caused approximately 100% larval mortality after 72 h, and EC50=328.3μM (Figure 1B). In the body length test, compared with the control group, the 400 μM lupeol group showed decreased body length (Figure 1C). In the heart rate test, the heart rate was decreased in the 200 μM lupeol group (Figure 1D). Based on the results, we found that lupeol was toxic to zebrafish larvae at concentrations of more than 200 μM. Thus, we selected lupeol concentrations of 25, 50, and 100 μM for further zebrafish experimentation.

Previous studies have shown that zebrafish larvae grown in 10 μg/mL LPS until 3 dpf exhibit an obvious systemic inflammatory response and oxidative stress [25, 26]. In this study, we exposed 9-10 hpf zebrafish larvae to 10 μg/mL LPS until 3 dpf as a liver injury model (Figure 1E). Liver injury is the pathological basis of various hepatic diseases, including liver cell degeneration, necrosis and inflammatory cell infiltration. We examined the hepatoprotective effects of lupeol on LPS-induced ALI in zebrafish. H&E staining of liver sections revealed that LPS caused hepatocyte degeneration, and lupeol ameliorated the restoration of liver tissue structures in zebrafish larvae (Figure 1F).

These results suggested that lupeol antagonized liver injury by attenuating hepatic apoptosis. Moreover, the effective concentrations of lupeol were in the range of 25-100 μM, among which 100 μM was the most effective.

### Lupeol inhibits liver damage caused induced by LPS/D-GalN in mice

**Figure 2:**
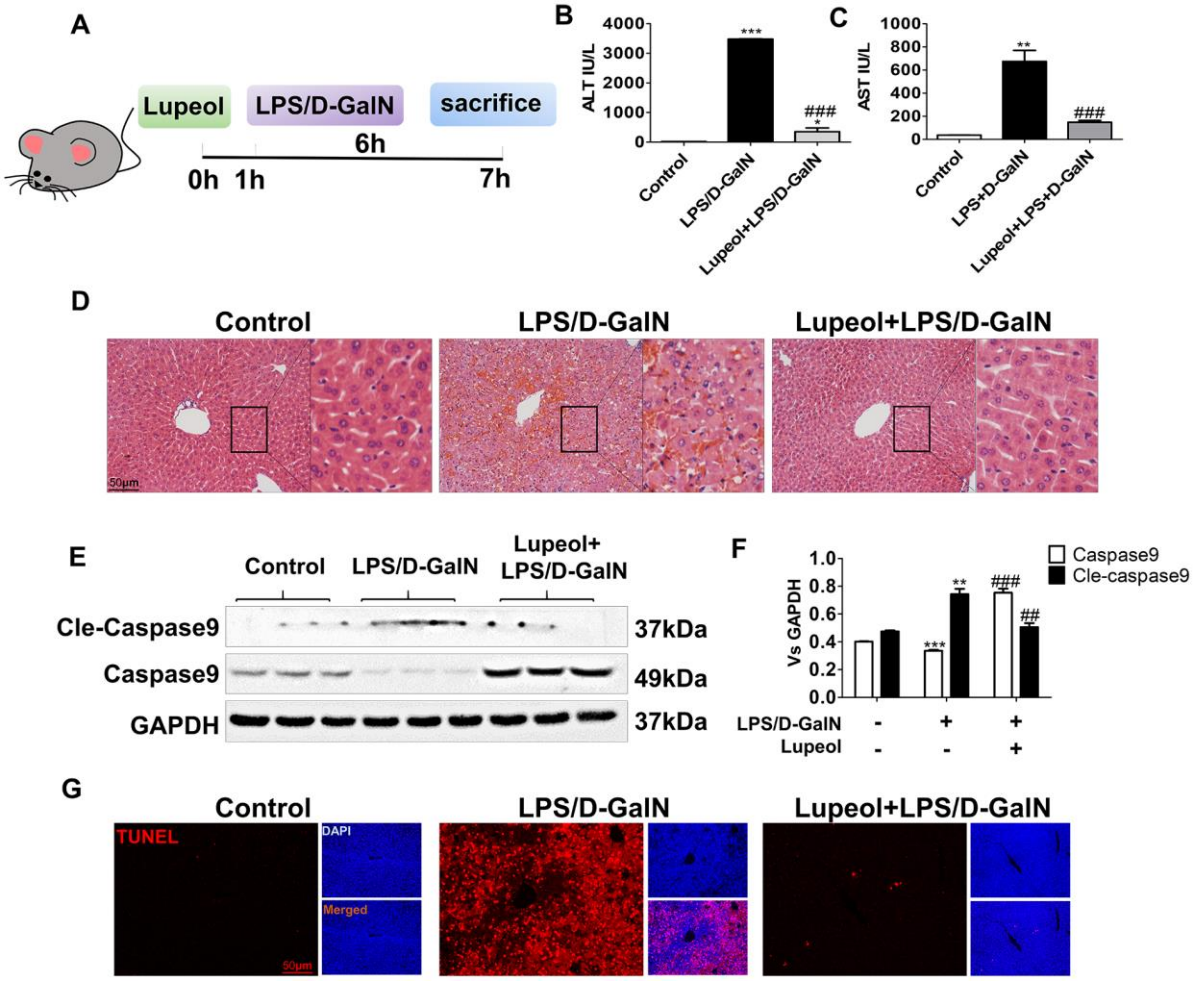
**Lupeol alleviates LPS/GalN-induced liver injury in mice.** (**A**) Diagrammatic sketch mice modeling and lupeol administration. (**B**, **C**) Changes in serum ALT and AST. (**D**) H&E staining was used to detect the liver histopathological changes. (**E**, **F**) Expression of caspase 9 and cleaved-caspase 9 protein in mice. (**G**) TUNEL staining of paraffin liver sections in control mice and mice treated with LPS/GalN or lupeol. Data are expressed as the mean ± SEM, n=3-6 per group, *P<0.05, **P<0.01, ***P<0.001, control group vs other groups. #P<0.05, ##P<0.01, ### P<0.001. Figures are magnified as 200x, bar=50μm.

LPS/D-GalN coinjection is a common method to establish an ALI model in mice, as D-GalN increases the sensitivity of rodents to LPS-induced hepatotoxicity [27]. To further verify the role of lupeol in ALI, an LPS/D-GalN-induced liver injury model was used (Figure 2A). As demonstrated by H&E staining, we found that the livers of the model groups exhibited diffuse necrosis, swollen hepatocytes and severe hemorrhage compared to those of the control group, while lupeol reduced liver necrosis (Figure 2D). In addition, serum ALT/AST levels were significantly increased in mice under LPS/D-Gal coinjection, while the levels were similar to those of the control group after lupeol treatment (Figure 2B, 2C).

Caspase 9, the initiator of apoptosis, activates the mitochondrial apoptosis pathway. Caspase 9 can combine with cytochrome c and the signal connector molecule Apaf-1 to form a complex after mitochondria release cytochrome c [28]. At the same time, it is cut into cleaved caspase 9. Cleaved caspase 9 further activates the downstream apoptosis executor caspase 3 to carry out a series of cascade reactions, which leads to apoptosis. In our study, the expression of caspase 9 was decreased, while cleaved caspase 9 was increased after LPS/D-GalN administration. After lupeol treatment, the expression of caspase-9 was increased, but the expression of cleaved caspase 9 was decreased (Figure 2E, 2F). Moreover, we used TUNEL staining to detect hepatocyte apoptosis in liver tissues and found that a large area of hepatocyte apoptosis occurred in the liver with LPS/D-GalN coinjection, while a significant reduction in hepatocyte apoptosis appeared with lupeol pretreatment (Figure 2G Supplementary Figure 1B).

These data showed that administration of LPS/D-GalN induced fatal ALI in mice. Lupeol inhibited aggravated liver injury by reducing hepatocyte apoptosis and decreasing the expression of cleaved caspase 9.

### Lupeol restrains LPS/D-GalN-induced inflammatory responses in zebrafish and in mice

**Figure 3:**
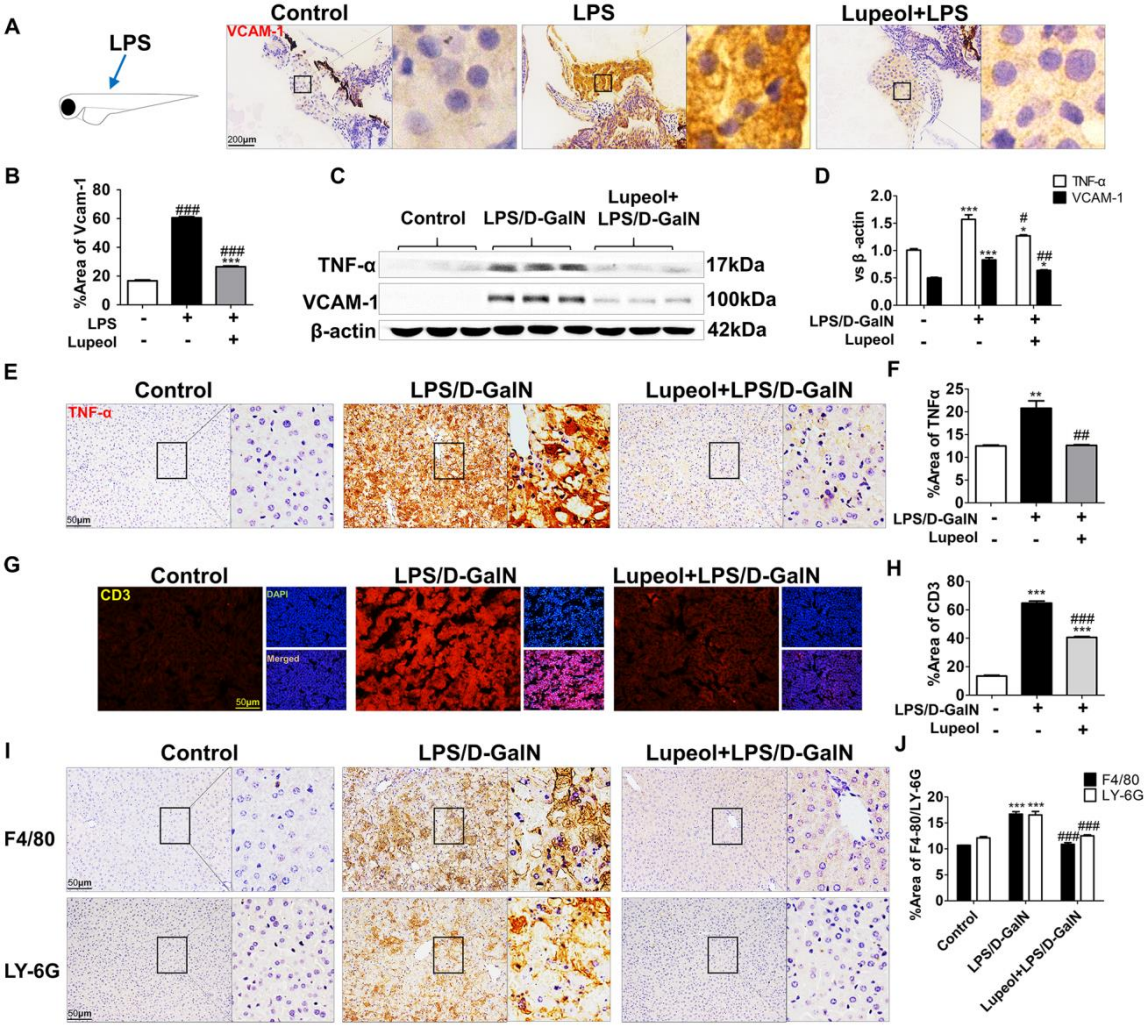
**Lupeol restrains LPS/GalN-induced liver inflammation.** (**A**) Immunohistochemistry staining of VCAM-1 in zebrafish, n=20, figures are magnified as 400x, bar=200μm. (**B**) Quantitative analysis of VCAM-1, data are represented as mean ± SEM. (**C**) Expression of TNF-α and VCAM-1 in mice. (**D**) Quantitative analysis of TNF-α and VCAM-1 protein. (**E**, **F**) Immunohistochemistry analysis for TNF-α location and expression in mice. (**G**, **H**) Immunofluorescence analysis for CD3 expression in mice. (**I**, **J**) Immunohistochemistry staining of F4/80 and LY-6G in mice and quantification of F4/80 and LY-6G expression. Data are shown as the mean ± SEM, n=3-4 group, *P<0.05, **P<0.01, ***P<0.001, control group vs other groups. #P<0.05, ##P<0.01, ### P<0.001. Figures are magnified as 200x, bar=50μm.

LPS/D-GalN induces liver injury mainly through inflammation. Generally, inflammation is caused by the release of TNF-α from natural killer cells, T lymphocytes and macrophages [29]. Vascular cell adhesion molecule-1 (VCAM-1), an adhesion molecule activated by TNF-α, recruits leukocytes to injury sites to initiate inflammatory responses [30, 31]. We found that lupeol treatment effectively reduced the expression of VCAM-1 in zebrafish (Figure 3A, 3B). Similarly, we also found that lupeol reduced VCAM-1 in mice (Figure 3C, 3D). Furthermore, we found that administration of lupeol significantly decreased the protein expression of TNF-α (Figure 3C–3F).

F4/80, a marker of macrophages, Ly-6G, a marker of neutrophils, and CD3, a marker of T cells, were detected by immunochemical staining in the livers of the mice. We found that compared with those in the control group, the numbers of cells with positive F4/80, Ly-6G and CD3 staining in the liver of the LPS/D-GalN-treated group were significantly increased, while pretreatment with lupeol inhibited the significant increase in immune cell infiltration (Figure 3G–3J). Collectively, these data indicated that lupeol reduced inflammatory responses in ALI.

### Lupeol attenuates LPS/D-GalN-induced oxidative stress in zebrafish and in mice

**Figure 4:**
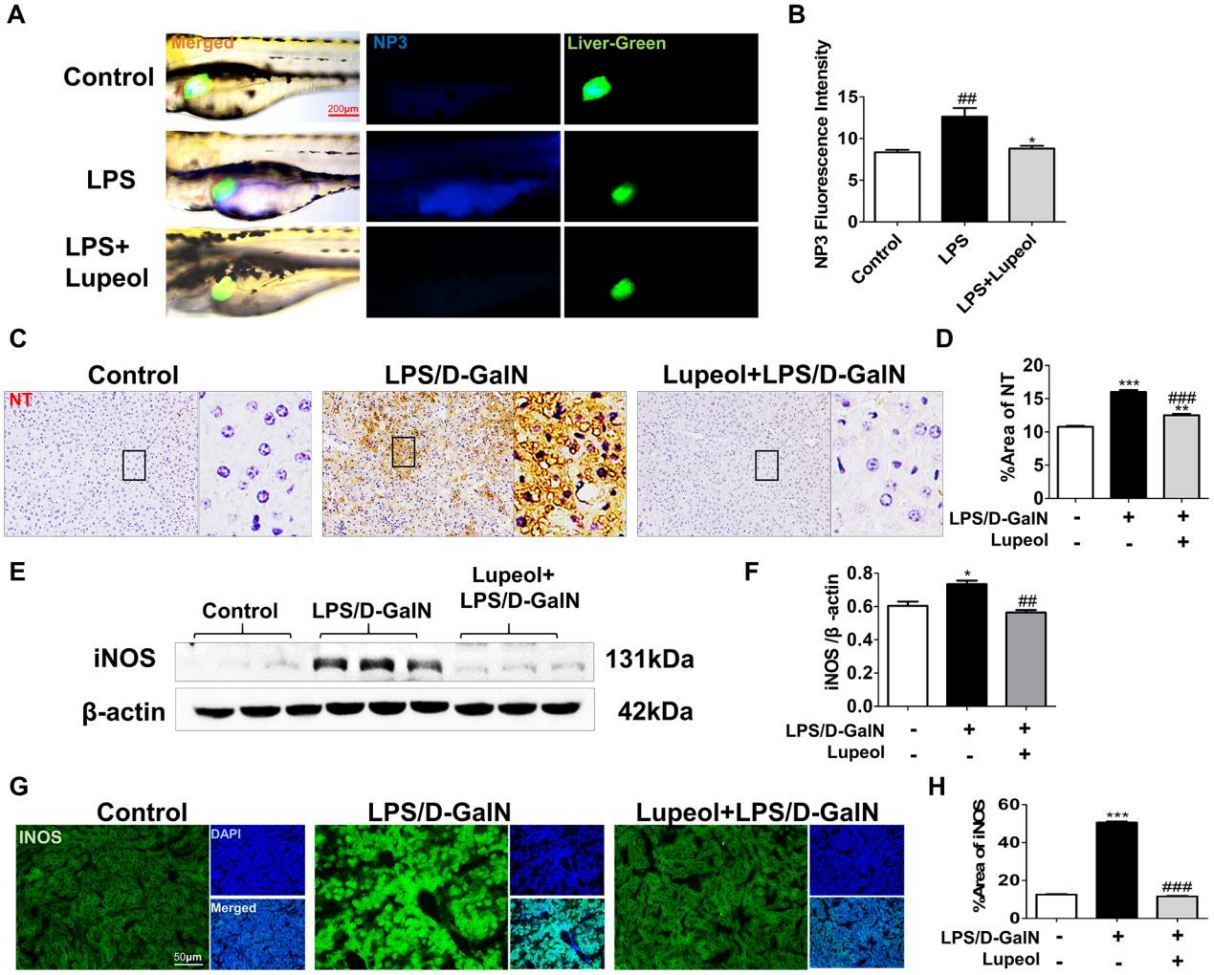
**Lupeol reduces oxidative stress in zebrafish and in mice with ALI.** (**A**) Fluorescence micrographs of ONOO^-^ generation in the control zebrafish larvae and the zebrafish larvae treated with LPS or lupeol. N=3-5, figures are magnified as 400x, bar=200 μm. (**B**) Quantification of the amounts and distribution of ONOO^-^. Data are exhibited as mean ± SEM. (**C**, **D**) NT immunohistochemical staining of mice livers and its quantitative analysis. (**E**, **F**) Western blots and quantitative results for iNOS. (**G**, **H**) Immunofluorescence staining and quantification for iNOS. All data are shown as the mean ± SEM, n=3-4 group, *P<0.05 vs control group, #P<0.05 vs model group. Figures are magnified as 200x, bar=50μm.

Peroxynitrite (ONOO^-^) is a highly reactive oxygen species. Abnormal regulation of ONOO^-^ in living systems is associated with diseases such as inflammatory conditions, auto-immune, and neurodegenerative diseases [32]. To detect the distribution and levels of reactive nitrogen species, we used liver-specific EGFP transgenic zebrafish and NP3, a fluorescent dye that can penetrate the cell membrane and blood-brain barrier, is suitable for detecting the level of ONOO^-^ in living cells. After the larvae were incubated with the NP3 fluorescent probes for 10 min, we collected and photographed the larvae. When the level of ONOO^-^ in the cell is higher, the blue fluorescence is stronger. We found that the level of RNS in zebrafish livers increased notably with LPS administration but decreased with treatment with 100 μM lupeol (Figure 4A, 4B).

Nitrotyrosine (NT) is an important tool for the detection of newly nitrosylated proteins, the determination of protein nitrosylation, and the measurement of nitrosylated protein levels in tissues and samples. Nitrated proteins were found to be significantly elevated in the livers of mice with LPS/D-GalN-induced injury. However, nitrated proteins decreased after lupeol treatment (Figure 4C, 4D). INOS levels increased in the livers of mice after LPS/D-GalN coinjection but decreased in the livers of mice pretreated with lupeol (Figure 4E–4H).

Generally, these results showed that lupeol could alleviate LPS/D-GalN-induced liver injury by inhibiting oxidative stress and partially by improving the antioxidant capacity *in vivo*.

### Lupeol ameliorates LPS/D-GalN-induced liver injury through the TGFβ1 and Nrf2 pathway

**Figure 5:**
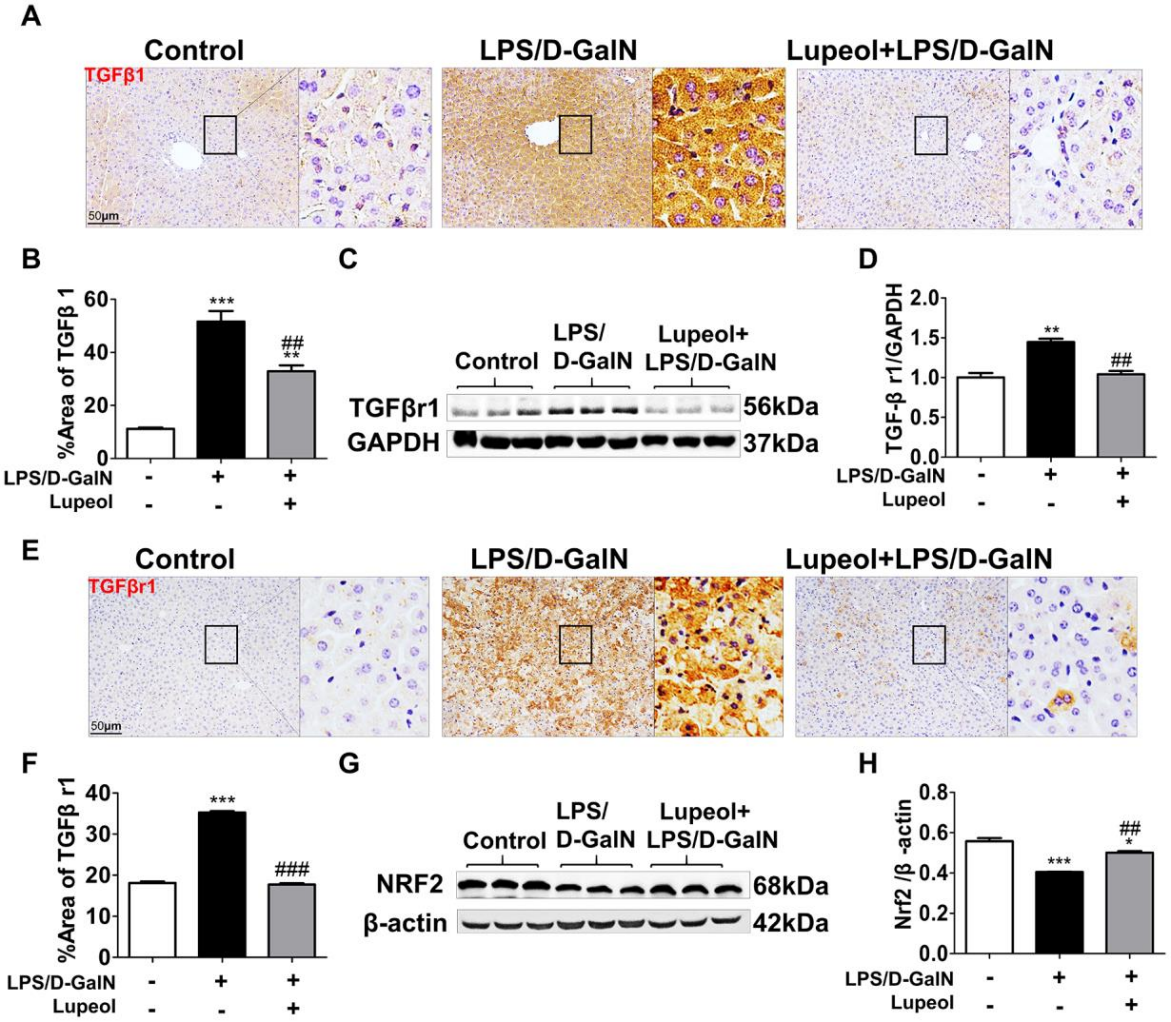
**Lupeol downregulates TGFβr1 and upregulates Nrf2 pathway in mice with ALI.** (**A**, **B**) Immunohistochemistry staining and quantitative analysis of TGFβ1. (**C**) Western blot analysis for the expressions of TGFβr1. (**D**) Quantitative analysis of TGFβr1 protein. (**E**, **F**) Immunohistochemistry analysis and quantification for detecting the expressions of TGFβr1. (**G**, **H**) Western blots and quantitative results for Nrf2. All data are shown as the mean ± SEM, n=3-4 group, *P<0.05 vs control group, #P<0.05 vs model group. Figures are magnified as 200x, bar=50μm.

Due to the pivotal role of the TGFβ1 pathway in the regulation of numerous cell processes, including extracellular matrix formation, cell proliferation, growth development, inhibition, and cell death [33], we detected whether lupeol treatment could affect the TGFβ1 pathway. Herein, lupeol efficiently repressed the protein expression of TGFβ1 in mice with LPS/D-GalN-induced ALI (Figure 5A, 5B).

Additionally, TGFβ1 transduces its signal by binding a heterotetramer receptor complex made up of TGF-β receptor 1 (TGFβr1) and TGF-β receptor 2 (TGFβr2). We found that lupeol could obviously decrease the protein expression of TGFβr1 in mice coinjected with LPS/D-GalN (Figure 5C–5F).

Moreover, the Nrf2 pathway plays a major role in regulating the expression of numerous antioxidant enzymes [34]. In our research, we found that lupeol promoted the expression of Nrf2, which was inhibited by LPS/D-GalN-induced liver injury (Figure 5G, 5H). In conclusion, lupeol could cure ALI by inhibiting TGFβ1 expression and promoting Nrf2 expression.

## DISCUSSION

ALI, a common disease that has high mortality, a poor prognosis, and no effective treatment, is associated with increased oxidative stress and inflammatory responses [5]. In this study, the principal findings obtained from histological and molecular studies are as follows. We found that lupeol could alleviate LPS/D-GalN-induced ALI by inhibiting inflammation caused by immune cell infiltration and oxidative stress resulting from RNS/ROS accumulation. Moreover, lupeol could protect the liver from LPS/D-GalN-induced injury by reducing the expression of TGFβr1 and increasing Nrf2, indicating that the TGFβr1-Nrf2 pathway was a possible target of lupeol.

Lupeol, which is present in diverse species of the plant kingdom, exhibits a spectrum of pharmacological activities against various diseases, such as cancer, microbial infections, arthritis, cardiovascular disease, diabetes, renal disease and liver disease [35–40]. A recent study demonstrated that lupeol ameliorated LPS/D-GalN-induced liver injury by inhibiting IRAK-mediated TLR inflammatory signaling [41]. In our study, we found that lupeol restored LPS/D-GalN -induced liver injury by repressing RNS/ROS accumulation and immune cell infiltration and that lupeol relieved ALI by regulating the TGFβ1 signaling pathway.

TGFβ1, which is involved in various stages of liver disease progression, plays a significant role in initial liver injury, liver inflammation, fibrosis and liver cancer [42, 43]. A previous study observed that Kupffer cells expressed high steady-state levels of TGFβ mRNA in CCl_4_-injured rat livers and that antisense S-oligodeoxynucleotides can restore CCL_4_-induced liver injury by downregulating TGFβ production [44]. As TGFβ plays a major role in liver regeneration, a recent study found that inhibition of TGFβR1 activity alleviated CCL_4_-induced intoxication by facilitating liver regeneration [45]. Moreover, a current study discovered that TGFβ signaling was activated in acute injury and that inhibition of TGFβR1 signaling reduced hepatocellular senescence by improving liver regeneration, function and outcome in acute liver injury [46]. In the present study, we further confirmed that TGFβ signaling was activated by LPS/GalN-induced liver injury. In addition, clinical trials of TGFβR1 or TGFβ inhibitors in human tumors are underway [46, 47]. Thus, our experiments provide a theoretical basis for the application of TGFβR1 or TGFβ inhibitors in acute liver damage and liver failure in humans.

Nrf2, considered a potential therapeutic target for preventing liver injury, plays an important role in the regulation of inflammation and oxidative stress [34, 48]. A recent study demonstrated that Nrf2 pathway was inhibited by a hepatotoxic drug matrine and accompanied with the activation of the ROS-mediated mitochondrial apoptosis pathway [49]. Moreover, another study showed that Licochalcone A has a hepatoprotective effect during LPS/GalN-induced liver injury by inducing the activation of Nrf2 and QSTM1(p62) signals and promoting autophagy via AMP-activated protein kinase (AMPK) signaling [50]. Furthermore, Nrf2 plays a major role in ameliorating various oxidative stress-associated diseases and exerts significant function in the antioxidant system, a recent study demonstrated that adropin reduced liver injury in nonalcoholic steatohepatitis by upregulating the expression of glutamate-cysteine ligase catalytic subunit(GCLC), glutamate-cysteine ligase regulatory subunit(GCLM) and glutathione peroxidase 1(Gpx1), dependent on Nrf2 transcriptional activity and increasing GSH levels [51]. Former study demonstrated that TGFβ1 induces Nrf2 mediated HO-1 expression and antioxidant response element activity in human aortic smooth muscle cells [11]. In this study, our results shown that lupeol administration improved hepatic antioxidant capacity to alleviate acute liver injury associated with Nrf2 up-regulation.

**Figure 6:**
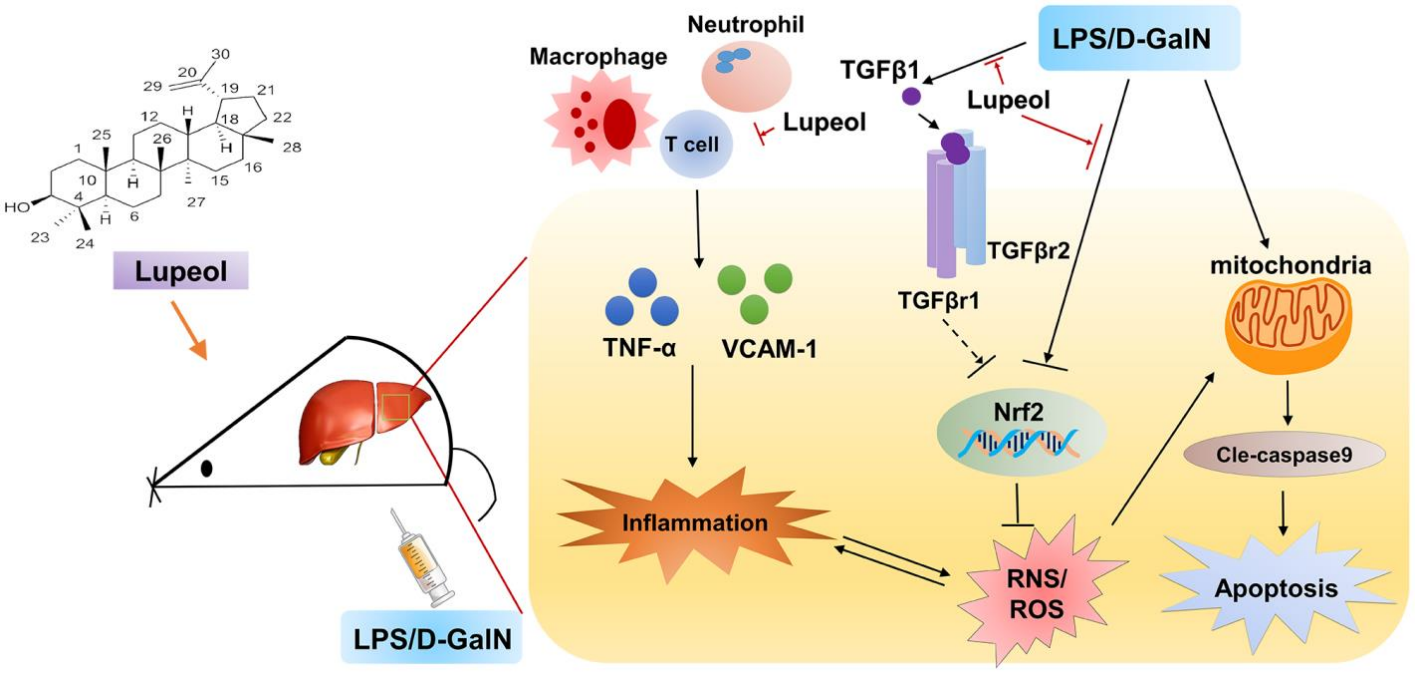
**Graphical abstract: A schematic diagram describing the protective mechanisms of lupeol against LPS/GalN-induced acute liver injury.**

In summary, our results demonstrate that lupeol attenuates LPS/GalN-induced ALI by restraining hepatic inflammatory and oxidative stress and inhibits activation of TGFβ1 induced by LPS/GalN administration (Figure 6). However, our study does not address whether lupeol is effective for acute liver injury and failure in humans, and further human safety and efficacy studies are required. Furthermore, but there is no further experimental study on the effect of TGFβ1 on acute liver disease. Therefore, we will further clarify the signal transduction relationship between TGFβR1 and Nrf2 in our future research.

## MATERIALS AND METHODS

### Animals and ethics statement

Wild-type (WT) mice with a C57BL/6 background between 9 and 12 weeks of age were genotyped, housed in a temperature-controlled animal facility with alternating 12:12 h light-dark cycles and fasted overnight before surgery but allowed to drink water ad libitum. All the procedures used in the animal studies were approved by the National Institutional Animal Care and Ethical Committee of Southern Medical University. All the experimental methods performed in this study were in accordance with the approved guidelines.

### ALI model and lupeol intervention

LPS (*E. coli*, L2630) and D-GalN (G0500) were purchased from Sigma (St. Louis, MO), and lupeol (B21602) was obtained from Shanghai Yuanye Bio-Technology. For the ALI model, male mice were intraperitoneally (i.p.) injected with LPS (20 μg/kg) 15 min before injection of D-GalN (700 mg/kg). Mice in drug treatment group was taken Lupeol (80 mg/kg each mouse) by intragastric administration 1 h before LPS and D-GalN treatment. Mice were sacrificed 6 h after LPS and D-GalN administration.

### Zebrafish maintenance and treatment

WT and Tg(lfabp10α–EGFP) adults and larvae were maintained with a light: dark period of 14:10 h at 28° C. Embryos were gathered and developed in chorion water (0.5 mg/L methylene blue) for up to 5 days post fertilization (dpf) at 28.5° C. All zebrafish procedures were approved by the Institutional Animal Care and Use Committee of Southern Medical University.

Zebrafish larvae at 9-10 hpf were stochastically divided into six groups: the control group, the 0.1% DMSO group, the model group, and 3 therapy groups. In the control group, embryos were raised in fish water. In the 0.1% DMSO group, embryos were raised in 10 μg/mL LPS with 0.1% DMSO. In the model group, embryos were incubated in 10 μg/mL LPS until 3 dpf. In the therapy groups, lupeol was made soluble in dimethyl sulfoxide (DMSO) and diluted to 25 μM, 50 μM, and 100 μM with fish water. In addition, lupeol was added to the fish water 1 h before incubation in 10 μg/mL LPS. The larvae were raised in 6-well plates at a density of 30 larvae per well for approximately 61-62 h.

### Histological analysis

Liver tissues or zebrafish larvae were fixed in 4% PFA overnight at 4° C, dehydrated, soaked in xylene, and embedded in paraffin in sequence and then sliced into 4 μm sections. Paraffin sections were dewaxed with xylene, dehydrated with different concentrations of ethanol, stained with hematoxylin and eosin, dehydrated, cleared, sealed, and finally imaged under a light microscope (Nikon Eclipse Ni-U; Nikon, Tokyo, Japan).

### Serum alanine aminotransferase (ALT) and aspartate aminotransferase (AST) measurements

Serum was acquired by centrifugation of blood samples at 3000 g for 15 min, and AST and ALT activities were measured using an Alanine Aminotransferase Assay Kit (Nanjing Jiancheng Bioengineering Institute, C009-2-1) and an Aspartate Aminotransferase Assay Kit (Nanjing Jiancheng Bioengineering Institute, C010-2-1) according to the manufacturer’s instructions on a microplate reader at 510 nm.

### Immunofluorescence and immunochemical staining

Liver tissues were collected and routinely embedded in OCT. Frozen liver samples were sliced into 14 μm sections. For immunofluorescence staining, after being washed with PBS 3 times, the sections were penetrated with methanol at -20° C for 10 min and sealed with 5% goat serum at room temperature for 1 h. Finally, frozen liver sections were stained with CD3 (Affinity, AF5405) and INOS (Abcam, ab178945) overnight at 4° C. After extensive washing, the frozen sections were incubated with the respective fluorescent secondary antibodies. Finally, the nucleus was stained with DAPI for 10 min.

For immunochemical staining, paraffin sections (4 μm) were first dewaxed in xylene I, II, and III and then rehydrated in 100%, 95%, 90%, 80%, and 70% ethanol. Then, the samples were boiled in 1X sodium citrate, maintained at a sub-boiling temperature for 10 min to repair antigen and cooled to room temperature. Afterwards, endogenous peroxidase enzyme was inactivated using 3% H_2_O_2_ in methanol for 10 min in the dark at room temperature. After blocking nonspecific binding with 5% goat serum at room temperature, the sections were stained with antibodies against F4/80 (Affinity, DF2789), NT (Cell Signaling Technology, 9691S), TNF-α (Abcam, ab1793), TGFβr1 (Abcam, ab31013), and TGFβ1 (HuaAn, H1113) overnight at 4° C. The next day, sections were incubated with the respective biotinylated secondary antibodies. Positive staining was visualized using DAB. The reaction was stopped in ice water. Then, the samples were counterstained with hematoxylin, dehydrated, paraffinized and finally mounted and sealed with neutral gum. The dyed sections were photographed with an optical microscope (Nikon Eclipse Ni-U; Nikon, Tokyo, Japan).

### TUNEL assay

The TUNEL reaction was used to detect hepatocyte apoptosis in liver tissue by using frozen sections and the *In Situ* Cell Death Detection Kit (Roche). The cryosections were immersed in 0.01% Triton X-100 diluted in PBS for 10 min, washed with PBS, incubated with a 1:10 TUNEL working solution in a dark environment at 37° C for 1 h and washed 3 times with PBS. Then, DAPI was used to stain nuclei in the dark at room temperature for 5 min, and the samples were washed 3 times with PBS. The dyed cryosections were immediately photographed with an optical microscope (Nikon Eclipse Ni-U; Nikon, Tokyo, Japan).

### Fluorescent probe detection

The fluorescent probe NP3 (FYRK-FP-01-001KY) was used to detect ONOO^-^. NP3 was used to measure RNS. At 3 dpf, live larvae were immediately transferred into 24-well plates and incubated with a 10 μM solution at 28° C in the dark for 10 min. Then, the fluorescence distribution of NP3 was visualized with a bright-field dissecting microscope (Nikon Eclipse Ni-U; Nikon, Tokyo, Japan).

### Western blot analysis

Liver tissues were sonicated in ice-cold RIPA lysis buffer cell lysis buffer (Sigma) containing a phosphatase inhibitor cocktail (Sigma) and a protease inhibitor cocktail (Sigma). Protein concentrations were determined by a quantitative BCA assay. A total of 50 μg of protein was used for immunoblotting. Primary antibodies against INOS (1:1000, Abcam, ab178945), Nrf2 (1:1000, Proteintech, 16396-1AP), TGFβr1 (1:1000, Abcam, ab31013), caspase 9 (1:1000, Cell Signaling Technology, 9504P), cleaved caspase 9 (1:1000, Cell Signaling Technology, 9509P), TNF-α (1:1000, Abcam, ab1973), VCAM-1 (1:1000, HuaAn, HK0612), β-actin (1:2000, Affinity, T0022) and GAPDH (1:2000, Cell Signaling Technology, 2118S) were applied in the study.

### Statistical analysis

The numerical results are shown as the mean ± standard deviation (SD). All statistical analyses were carried out with GraphPad Prism version 5.01 software and SPSS 20.0. One-way ANOVA or an unpaired t-test was used for statistical analysis, and Tukey’s multiple comparison test was used for the appropriate experiments. P-values less than 0.05 were considered statistically significant.

## Supplementary Material

Supplementary Figure 1
