# Supplementary material for: Lupeol ameliorates LPS/D-GalN induced acute hepatic damage by suppressing inflammation and oxidative stress through TGFβ1-Nrf2 signal pathway
Source: Aging (Albany NY). 2021 Mar 11;13(5):6592–605. doi: 10.18632/aging.202409 (PMC7993700; doi:10.18632/aging.202409)
Supplement: Supplementary Figure 1 [file aging-13-202409-s001.pdf]

SUPPLEMENTARY FIGURE

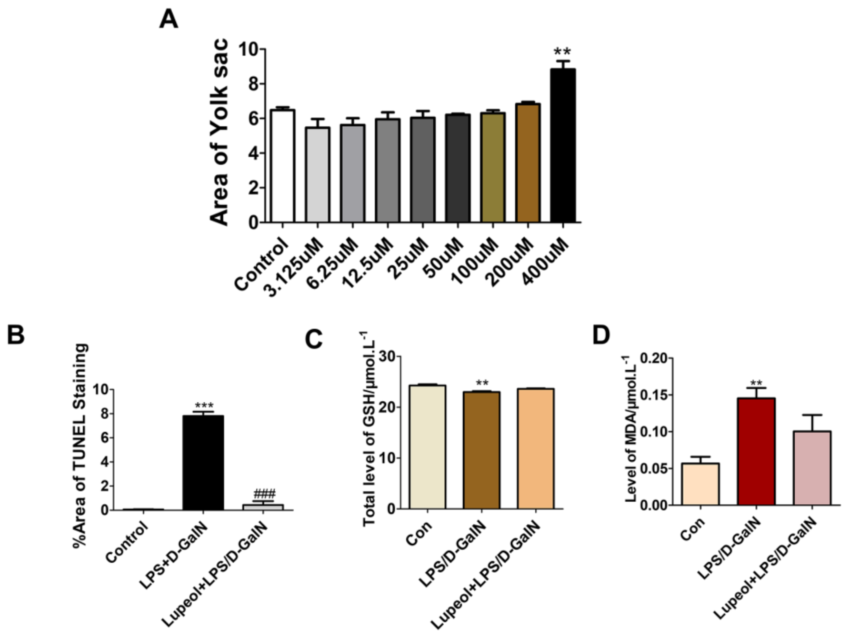

Supplementary Figure 1. Quantification of yolk sac, TUNEL staining, and detection GSH and MDA Level. (A)Quantitative analysis of yolk sac, n=10, data are represented as mean ± SEM. (B)Quantitative analysis of TUNEL staining of the control, LPS+D-GalN and Lupeol treatment group. (C and D) Changes in serum GSH and MDA. Data are shown as the mean ± SEM, n=3-4 group, *P<0.05, **P<0.01, ***P<0.001, control group vs other groups. #P<0.05, ##P<0.01, ### P<0.001.
